# Role of AuNPs in Active Food Packaging Improvement: A Review

**DOI:** 10.3390/molecules27228027

**Published:** 2022-11-18

**Authors:** Hamed Ahari, Mostafa Fakhrabadipour, Saeed Paidari, Gulden Goksen, Baojun Xu

**Affiliations:** 1 Department of Food Science and Technology, Science and Research Branch, Islamic Azad University, Tehran 1477893855, Iran; 2 Department of Food Science and Technology, Qeshm Branch, Islamic Azad University, Qeshm 7953163135, Iran; 3 Department of Food Technology, Vocational School of Technical Sciences at Mersin Tarsus Organized Industrial Zone, Tarsus University, Mersin 33100, Turkey; 4 Food Science and Technology Program, Department of Life Sciences, BNU-HKBU United International College, Zhuhai 519087, China

**Keywords:** antimicrobial effects, food packaging, gold nanoparticles, nanotechnology, shelf life

## Abstract

There is a worldwide concern about food loss due to reduced shelf life among food science researchers. Hence, it seems that any techniques contributing to improved food packaging are most welcome in the food sector. It has been demonstrated that the administration of nanotechnology-based techniques such as metal-based nanoparticles can fade away the unresolved obstacles in shortened shelf life and environmental concerns. Along with substantial signs of progress in nanoscience, there is a great interest in the usage of green synthesis-based methods for gold nanoparticles as the most advantageous metals, when compared to conventional chemistry-based methods. Interestingly, those aforementioned methods have significant potential to simplify targeted administration of gold nanoparticles due to a large surface-volume ratio, and diminished biohazards, aimed at increasing stability, and induction of anti-microbial or antioxidant properties. However, it is necessary to consider the hazards of gold nanoparticles including migration for food packaging purposes.

## 1. Introduction

It has been demonstrated that there is a worldwide concern about the growing rate of food loss related to perishable food [1,2]. Results acquired from those surveys depict that expiring on the shelves of food stores makes problematic challenges leading to food loss [3]. In other words, food spoilage is accelerated by several microbial activities, and enzymatic activation of oxygen-driving processes [4]. Accordingly, these issues are addressed in imposing a financial burden on the economy, health system, environment, and even on recycling industries [5]. Thereafter, it seems legal to move forward to the establishment of any technological approaches aimed at the improvement of food quality.

Proper food packaging (FP), which is traced back to the 18th century, can play an indispensable role in the prolongation of shelf life of food products [6], and also preserves nutritional values during various stages such as transport, distribution, and storage [3]. Additionally, reduction of microbial contamination especially during harvesting, processing, slaughtering, and packaging steps in developing countries, is of high practical and industrial importance [1,7,8]. 

**Figure 1:**
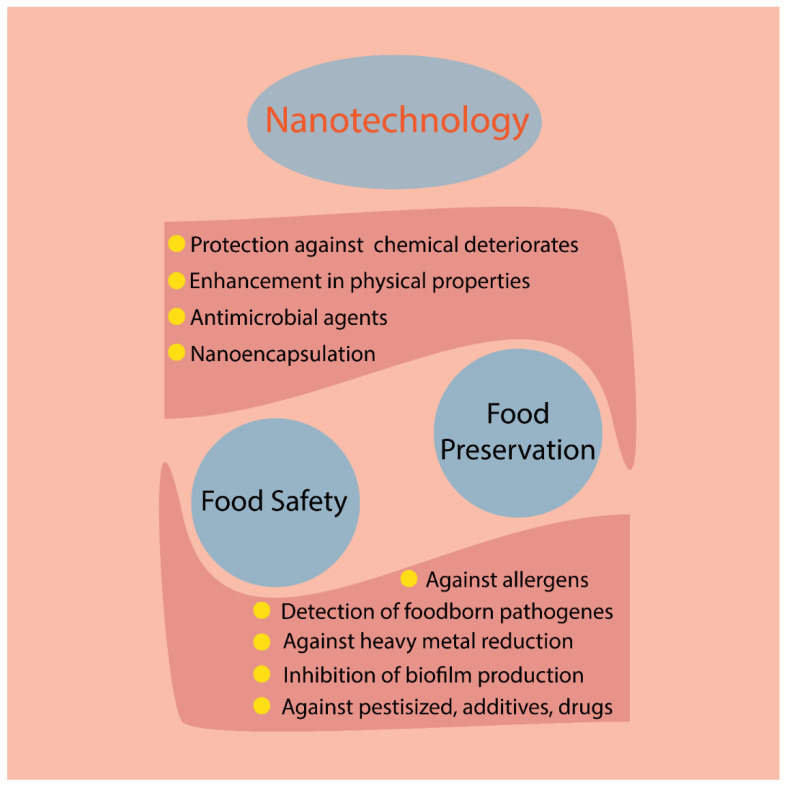
A schematic presentation of nanotechnology applications in daily life.

As mentioned earlier, there is an imperative need for signs of progress toward more efficient biotechnology-based applications in FP. A wide range of technologies including biomaterials and nanotechnology are utilized in FP [9]. Many researchers have focused on the employment of nanotechnology for food packaging purposes (Figure 1) [10].

Nanotechnology in FP has a wide array of advantages, including making a barrier between the content of the food and exterior atmosphere [11], and assuring prevention of possible post-contamination of food, aimed at potential production and processing of healthier, safer, and high-quality foods [12]. There is a wide range of nanoparticles that are extensively employed in food packaging systems with distinct purposes including antibacterial [13], gas scavenging [14], antioxidant [15], and barrier properties [16] while considering food quality detectors as smart food packaging systems [17]. Metal nanoparticles (e.g., Ag, Au, Cu, CuO, Zn, ZnO, TiO_2_), nano clay [18], nanoemulsions (e.g., essential oil of plants such as rosemary, ginger) metal-organic frameworks (MOF) [19] are known as the most employed nanoparticles in FP systems [13].

Amongst the existing nanoparticles, metal-based nanoparticles have attracted the attention of scientists to incorporate with the biomaterials to makes suitable packaging. Considering metal-based nanoparticles, gold nanoparticle (Au) has shown noticeable potential in improving food packaging systems from the viewpoint of antibacterial activity, improving barrier properties, using as biosensors, scavenging and so on. The incorporation of AuNPs into polymeric composites plays a crucial role in the active packaging of food. Recently, incorporation of AuNPs and grapheme oxide separately on PVA (polyvinyl alcohol) composite films on banana demonstrated antimicrobial activity against *Escherichia coli*. Banana shelf life was qualitatively enhanced with PVA-glyoxal-AuNPs nanocomposite for preserving food, validating its application in food packaging [18]. In another study, AuNPs conjugated with gallic acid (GA-AuNPs) were examined as an effective and promising strategy for reducing oxidative damage and physiological degradation of food products. The potential capacity to scavenge free radicals and green catalytic activity against microorganisms are features of the compound. Results showed that GA-AuNPs is the safest and most effective food packaging liner in the food business [13].

In this paper, the main aim is to provide a comprehensive review of the role of gold nanoparticles (AuNPs) in food packaging systems. In this regard, first, a brief discussion was presented considering the way nanoparticles are employed in FP systems; then, the synthesis of AuNPs, and their potential in FP systems are discussed.

## 2. Search Method

Data of this review study were collected through an online literature review (in the English language) in Scopus, PubMed, Cochrane, Science Direct, and Google Scholar databases/search engines by 8 keywords (including 5 main keywords: (Gold nanoparticle, Au nanoparticle, AND Food Packaging, AND Food Safety), and 3 complimentary keywords (Anti-bacterial, Anti-oxidant, Shelf life)) commencing from January 2011 until June 2021 time intervals. 

## 3. Synthesis of AuNPs

The modern era of AuNPs synthesis began over 150 years ago with the work of Michael Faraday, who was possibly the first to observe that colloidal gold solutions have properties that differ from bulk gold. Among the several forms of NPS created by biological systems, the gold ones are recognized as being the most compatible and non-toxic to the human body. Based on their particular characteristics and distinct surface functions, AuNPs have been used in food packaging. The simplicity of AuNP operation and functionalization results in a multi-platform in food packaging systems for antibacterial, gas scavenging, preventing biofilm formation, inhibiting gas permeability, and other purposes [19].

Along with tremendous development in the usage of AuNPs-based approaches in a variety of FP systems, there are growing concerns about the commercialization of conventional synthesis methods due to environmental or clinical hazards [20]. Over the past few decades, a wide range of solution-based approaches is employed to control size [21], shape [22], and surface functionality [23].

However, so far there is no available report regarding the nano-toxicity of biologically produced gold NPs (AuNPs) in vitro and in vivo. There are several reports regarding the synthesis of AuNPs via different chemical agents. For instance, citrate and gold salt are two famous components to produce gold nanoparticles (Equation (1)) [24]. Scientists showed that environmental condition including temperature affects the AuNPs dimension and their life cycle. It was reported that citrate and oxidation by-products (e.g., ACDC^2−^ (acetone dicarboxylate)) promote gathering and bridging Au(III) ions around the AuNPs seeds in the initial growth step [24].

Equation (1)

(1) $${2\mathrm{AuCI}}_{4}{}^{-}{+3\mathrm{Ctr}}^{3-}\;\to {\;2\mathrm{Au}+3\mathrm{ACDC}}^{2-}{+3\mathrm{CO}}_{2}↑{\;+8\mathrm{CI}}^{-}{+3H}^{+}$$

ACDC^2−^ can be converted to acetone at 100 °C according to Equation (2). However, past studies approved that the products from the Equation (2) can react with auric chloride and cause complete conversion to produce Au0 (Equation (3)) [24,25]. 

Equation (2)

(2) $${\mathrm{ACDC}}^{2-}{+2H}_{2}O\;\overset{100\;{}^{\circ}C}{\to }\;\mathrm{acetone}\;+\;2{\mathrm{CO}}_{2}↑{\;+2\mathrm{OH}}^{-}$$

Equation (3)

(3) $${4\;\mathrm{AuCI}}_{4}{}^{-}{+\;6H}_{2}O+3\;\mathrm{acetone}\;\;\overset{100\;{}^{\circ}C}{\to }\;{4\mathrm{Au}+9\mathrm{CH}}_{2}{O+12H}^{+}{+16\mathrm{CI}}^{-}$$

Summing up Equations (1)–(3), the following equation is obtained in which the effect of temperature ACDC^2−^ degradation is not considered:

Equation (4)

(4) $${2\mathrm{AuCI}}_{4}{}^{-}{+\mathrm{Ctr}}^{3-}{+2H}_{2}O\;\overset{100\;{}^{\circ}C}{\to }\;{2\mathrm{Au}+3\mathrm{CH}}_{2}{O+3\mathrm{CO}}_{2}↑{+8\mathrm{CI}}^{-}{+3H}^{+}$$

In another study, Equation (5) has been speculated at room temperature [26]:

Equation (5)

(5) $${6\mathrm{ACDC}}^{2-}{+22\mathrm{AuCI}}_{4}{}^{-}{+24H}_{2}O\;\overset{\mathrm{Room}\;\mathrm{temp}}{\to }\;{22\mathrm{Au}+6\mathrm{CH}}_{2}{O+3\mathrm{HCOOH}+21\mathrm{CO}}_{2}{+88\mathrm{CI}}^{-}{+54H}^{+}$$

In another research, it was shown that the polarity of the solvent affects the AuNPs production and their size [26,27]. It was shown that the smaller size of AuNPs was synthesized in the high polarity index of the ethanol/water solvent as the reaction medium, while the bigger size of AuNPs was synthesized in the low polarity index. Polarity can be calculated from Equation (6) [28].

(6) $$P^{\prime }=\sum p_{i}^{\prime }\phi_{i}$$

In which:

$p_{i}^{\prime }:$ the polarity index of solvent *i* in the mixture;

*ϕ_i_*: the volume fraction of solvent *i* in the mixture.

It is mandatory to diminish concerns about by-products as toxic chemicals and increase the stability of AuNPs. It has been demonstrated that it is necessary to consider safe and cost-effective methods and pave the path for the brisk emergence of green synthesis of AuNPs [29,30,31]. There is a relationship between the functions of AuNPs and life cycle evaluation on their environmentally friendly nature. It seems that employing green synthesis of AuNPs is of great prominence due to sustainable and renewable suppliers of chemical energy [32]. There are several benefits in green biosynthesis procedures to synthesize AuNPs, when compared with classical chemistry methods. Those aforesaid biological resources act as nano-factories with less reaction time, size-controlled capability, and high acceptability [33]. Among the types of NP production techniques, the biological method is widely accepted because the use of the living organisms in the production pathway is safer than other methods. In the rest, two techniques (using microbial strains and using leaf extract) for the green synthesis of AuNPs are discussed.

The aqueous reduction of gold salts with sodium citrate is one of the most typical techniques for the production of AuNPs [34], compared to the shape-controlled synthesis of AuNPs [35]. It has been demonstrated that reagents acting as reducing and stabilizing ones such as chitosan [36] or alginate can serve as the most efficient reagents for the fabrication of AuNPs in situ [37,38].

As a prime study conducted by Irshad A. Wani et al., they tried to evaluate the size and shape-dependent anti-fungal activity of various AuNPs [39].

Those recruited AuNPs were different in their reducing agents affecting particle size, morphology, and properties of nanoparticles under similar ultrasonic frequency circumstances and without using any stabilizer [40]. Accordingly, two types of AuNPs, including gold nanodiscs of the average diameter of 25 nm (tinchloride as reducing agent), and polyhedral (nanocrystal) structure of the average size of 30 nm (sodium borohydride as reducing agent), were used in this study. Gold nano-discs reveal a much higher surface area and stronger fungicidal activity through inhibition of H+-ATPase against *Candida albicans* (*C. albicans*) [41]. 

### 3.1. Biosynthesis of AuNPs Using Microbial Strains

Researchers have a strong interest in the use of microbial strains, such as bacterial, due to their manageability and cost-effectiveness [41], actinomycete [42,43], fungal [44], algal [45], and yeast strains [46], for development of AuNPs for food packaging purposes.

As it was pointed out before, in addition to the existence of phytochemicals in plants, there are other co-chemical substances synergized with phytochemicals agents such as citrate or cetyltrimethyl ammonium bromide (CTAB), being affected by photomutagenicity processes in AuNPs [47].

Results from the microbial study show that biosynthesis of AuNPs using microbial strains (such as *Shewanella oneidensis*) can be considered as highly efficient bio-reduction-based fabrication approaches [48].

It was reported that *Bacillus subtilis* could reduce Au^3+^ ions to AuNPs. The AuNPs size was reported between 5 and 25 nm inside the cell walls [49]. In an experimental study, Baker Syed et al. [50] investigated the synthesis of AuNPs from endophytic *Pseudomonas fluorescence* 417 inhabiting *Coffea arabica* L., and the assessment of their biological (anti-microbial) activities against clinically significant pathogens. AuNPs were confirmed by hyphenated techniques such as ultra violet (UV)-visible spectrophotometry (changing color of reaction from yellow to ruby red within 5 min of incubation), XRD, TEM, and FTIR (majorly in a spherical shape) [50]. Those AuNPs showed the most antibacterial properties against *P. aeruginosa* (MTCC 7903), *E. coli* (MTCC 7410), *S. aureus* (MTCC7443), *B. subtilis* (MTCC 121), and *Klebsiella pneumoniae* (*K. pneumoniae*) (MTCC 7407), respectively (results confirmed by colony forming unit (CFU) method and antibiogram tests). According to the results acquired from this study, the authors introduced such methods as efficient alternatives for the treatment of MDR strains [50].

In another study, Pourali and her colleagues employed two microorganisms (*Bacillus cereus* and *Fusarium oxysporum*) for the biosynthesis of AuNPs and compare their nanotoxicity under in vitro assays [51]. In this study, the authors added 100 microliters of HAuCl_4_ to the 100 mL of the separated microbial supernatant and incubated shaker incubator for 24 h at 37 °C and 200 rpm. Both strains showed success in AuNP synthesis. Results revealed that the AuNPs produced by both strains were hexagonal, spherical, and octagonal with sizes of around 20–50 nm and irregular contours.

Singh and his colleagues reported AuNPs production using *Rhodopseudomonas capsulata* and *Pseudomonas aeruginosa* bacteria [52] under pH values from 4 to 7. They reported that pH value was the important parameter to control the shape and size of AuNPs. The produced AuNPs had a spherical shape and a diameter between 20 and 80 nm at pH 6.5. They also reported that the spherical AuNPs with the size of 10–20 nm were produced at pH = 7 while many nanoplates were detected at pH = 4. *Shewanella* algae were found to reduce Au^3+^ ions forming 10–20 nm AuNPs extracellularly with the assistance of hydrogen gas [52,53]. On the other hand, the AuNPs have been prepared on the surface of bacteria as a result of incubation of the cells with Au^3+^ ions [54].

Fungi were capable of reducing the metal ions such as gold salt into their corresponding nanometals i.e., nanogold either in the intracellular or extracellular state depending on the position of the reduction enzymes. In other words, the nanoparticles were formed extra-cellular when the cell wall’s reduction enzymes were responsible for metal ions reduction and the reduction enzymes are secreted extra-cellular [55]. Sastry and coworkers have reported the extracellular synthesis of AuNPs by fungus *Fusarium oxysporum* and actinomycete *Thermomonospora sp*., respectively [56].

### 3.2. Biosynthesis of AuNPs Using Leaf Extract

According to the mentioned advantages for green biosynthesis of AuNPs, there is a growing trend in the usage of eco-friendly, cost-effective, non-hazardous, and sustainable methods for the synthesis of AuNPs [57]. As far as a wide arrow of studies is being devoted to green biosynthesis of nanoparticles, some of them believe that usage of several biopolymers as starch, chitosan, cyclodextrins, or even bacterial biomass [58], or environmental-based material such as olive-mill wastewater can be incorporated for reduction of AuNPs by phenolic compounds [59]. There are different industrial and laboratory approaches for the synthesis of leaf (or other parts of plants such as bark, root, or fruit) extract aimed at the usage for the biosynthesis of AuNPs [40,60]. They include (a) solvent-based extraction, (b) microwave-assisted extraction, (c) maceration extraction, and (d) ultrasound-assisted extraction [40]. In this part, several recently conducted studies aimed at the investigation on the green biosynthesis of AuNPs by leaf extract or marine algae, are summarized: Rajathi et al., aimed at investigation of anti-bacterial effects of AuNPs produced by *Stoechospermum marginatum* (kützing) as a brown alga in their experimental study [61]. Their results from transmission electron microscopy (TEM) indicated the presence of majorly spherical shapes in the nanoparticles with (photoluminescent features) and 45.92% elemental gold. Interestingly, hydroxyl groups were recognized as reduction agents (present in the diterpenoids of the brown seaweed), effectively acting against bacterial pathogens [61].

In another study by Chidambaram Jayaseelan et al. [62], anti-fungal properties of AuNPs produced by seed aqueous extract of *Abelmoschus esculentus* (green biosynthesis of AuNPs with crystalline nature), was investigated. *Puccinia graminis critic*, *A. flavus*, *A. niger*, and *C. albicans* were the fungal strains that were tested. The authors reported that all of the nanoparticles were spherical with a narrow size range of 45–75 nm. Anti-fungal activities were observed among all of the fungal strains, especially *C. albicans*, and *Puccinia graminis* with the most inhibition zone (18, and 17 mm, respectively.). The authors concluded that as this approach is a low-cost, and user-friendly approach (done at room temperature), it also leads to better control over their nanostructures. This approach could be significantly considered in nanomedicine, and food packaging industries (as nano-preservative agents).

Marjan Shariari et al. [63], designed their study aimed at biosynthesis, and characterization of anti-AuNPs produced by *Allium noeanum* Reut. ex Regel leaves. The authors declared that antioxidant compounds in fresh leaves can be responsible for reducing agents (and forming monodisperse particles), confirmed by Fourier-transform infrared spectroscopy (FTIR) techniques. Based on the results, not only synthesized AuNPs depicted excellent antioxidant potential against 1,1-diphenyl-2-picryl-hydrazyl (DPPH) and improved solubility, but they also revealed significant non-toxicity properties against human umbilical vein endothelial cells. 

Additionally, it is of high prominence to mention that those synthesized AuNPs indicated antibacterial activities against a wide array of Gram-negative or Gram-positive pathogens (*Streptococcus pneumoniae* (*S. pneumoniae*), *Bacillus subtilis* (*B. subtilis*), *Staphylococcus aureus* (*S. aureus*), *Staphylococcus saprophyticus* (*S. saprophyticus*), *Salmonella typhimurium* (*S. typhimurium*), *Pseudomonas aeruginosa* (*P. aeruginosa*), *Shigella flexneri*, and *E. coli* O157:H7). They introduced that leaves extract incubated with aqueous gold ions can be considered as the most reproducible and biocompatible methods [63].

Jayanta Kumar Patra et al. [64] in their experimental study aimed at the synthesis of AuNPs from aqueous extract of dried outer onion peel (onion peel as food waste materials), and assessment of their biological activities. It was reported that various water-soluble phenolic compounds and cysteine derivatives were responsible for the reduction of nanoparticles (45.42 nm in diameter). Promisingly, synergistic anti-bacterial (against five different foodborne pathogenic bacteria), anti-oxidant, and proteasome inhibitory potentials were reported. They declared that this aforesaid approach can be an eco-friendly, and nontoxic procedure being able to be conducted in one step.

Annamalai et al. [65] in their experimental study, aimed at the synthesis of AuNPs from *Euphorbia hirta* L. leaf extract, and assessment of their biological (anti-microbial) activities. They recruited surface plasmon resonance (SPR) for characterization and confirmation of synthesized AuNPs through changing the color of the extract from pale yellow to purple (nanoparticles with the size varying from 6 nm to 71 nm). Promisingly, anti-bacterial properties of those synthesized nanoparticles against several bacterial strains of *E. coli*, *P. aeruginosa*, and *K. pneumoniae* were reported at very low concentrations (tested concentrations varying from 1.25 to 200 g/mL) of nanopowders used for nanoparticles. Interestingly, complete inhibition on bacterial growth was reported at the highest concentration, and the most inhibited bacterial growth was reported for *K. pneumoniae* (94%). They declared that this aforesaid approach (especially with the usage of combinative aqueous and methanolic extracts of *Euphorbia hirta* L. leaf) can be an eco-friendly, a rapidly conducted, and efficient procedure (due to synergized agents), being potentiated for usage in a wide range of industries or clinical applications [65].

Several researchs are dedicated to the improvement of the antimicrobial effects of metal-based nanoparticles, as well as considerable indicators of success in the case of the combinational use of AuNPs with others. Exemplary examples of these studies are collected. One comparative experimental study conducted by Priyanka Singh et al. [66] was aimed at the evaluation of anti-inflammatory properties of spherical silver nanoparticles, and monodisperse hexagonal AuNPs. Those nanoparticles were produced by green biosynthesis from fruit extract of *Prunus serrulata* at 80 °C. In their study, nanoparticles were confirmed and characterized through visual observation (with the appearance of color change in reaction for silver, and AuNPs, including from ruby red to brown within 50 min, and from light ruby red to deep purple color within 30 s, respectively), UV-VIS FETEM, EDX, elemental mapping, FTIR, XRD, and DLS. Criteria for anti-inflammatory effects of both nanoparticles were inhibition of downstream NF-ĸB activation in macrophages [66]. Results of MTT assay from this study reported a significantly attenuated expression of inflammatory mediators such as nitric oxide (NO), prostaglandin E2 (PEG2), inducible nitric oxide synthase (iNOS), cyclooxygenase-2 (COX-2), and LPS-induced activation of NF-ĸB signaling pathway in LPS-induced RAW264.7 cells line as in vitro model. According to the results acquired from this study, the authors introduce the mentioned approach as an eco-friendly synthesis with biocompatible nature, being used as novel therapeutics for the prevention and cure of inflammation [66].

In another comparative experimental study done by R Geethalakshmi et al. [67], synthesis of gold and silver nanoparticles from *Trianthema decandra* L. root extract, and assessment of their biological (anti-microbial) activities were investigated. The shape of AuNPs was confirmed through FESEM (with the size for gold, and silver nanoparticles varying from 33 to 65 nm, and 36–74 nm, respectively), and the presence of metallic gold and silver in the nanoparticles was approved by EDX and FTIR. Phytochemical constituents were responsible for reducing agents. Both nanoparticles showed significant anti-bacterial/anti-fungal activities against *S. aureus* (MTCC 29213), *Streptococcus faecalis* (*S. faecalis*) (MTCC 0459), *Enterococcus faecalis* (*E. faecalis*) (MTCC 2729), *E. coli* (MTCC 443), *P. aeruginosa* (MTCC 1035), *Proteus vulgaris* (*P. vulgaris*) (MTCC 1771), *B. subtilis* (MTCC 121), *Yersinia enterocolitica* (*Y. enterocolitica*) (MTCC 840), and *C. albicans* (MTCC 183), especially in Gram-negative bacteria. Comparatively, the anti-bacterial properties of silver nanoparticles were more than AuNPs. But both of those fabricated nanoparticles showed notable anti-bacterial effects on *Y. enterocolitica*, *P. vulgaris*, *E. coli*, *S. aureus*, and *S. faecalis*. They declared that due to the smaller size of AuNPs (varying from 37.7 nm to 79.9 nm) compared to bacteria, adherence to the bacterial cell wall is simplified and accelerated, leading to bacterial cell death [67].

In another study conducted by S. Lokina et al. [68], promising results from an investigation on biological (anti-microbial) properties of AuNPs produced from *Punica granatum* fruit (pomegranate) extract, were reported. For characterization and confirmation of AuNPs (with the size ranging from ranges from 5 nm to 17 nm), UV-Vis, fluorescence, high-resolution AuNPs (HRTEM), XRD, FTIR, selected area electron diffraction (SAED), and thermogravimetric analysis (TGA), were used. Through SPR, it was found that auric chloride (chloroauric acid or AuCl4) is the responsible agent for the bio-reduction of AuNPs (through observing the color change from yellow to black in the reaction mixture) [68]. According to the minimum inhibitory concentration (MIC) assay, they reported an excellent anti-microbial activity of synthesized AuNPs against *C. albicans* (ATCC 90028), *A. flavus* (ATCC 10124), *S. aureus* (ATCC 25175), *Salmonella typhi* (ATCC 14028), and *Vibrio cholerae* (ATCC 14033). Additionally, according to the MTT assay, they reported excellent cytotoxic activity of synthesized AuNPs against HeLa cancer cell lines at different concentrations. Interestingly, the percentage of cancerous cell death was 98.9% in the presence of the highest dose of AuNPs (1000 μg/mL). They introduced the aforementioned green synthesis method as low cost, non-toxic, and eco-friendly [68]. Table 1 shows a wide range of studies, aimed at the investigation of green synthesis of AuNPs from plant sources. 

## 4. Potential Application of AuNPs in Food Packaging

Although the emergence of nano-biotechnology approaches is not a newly introduced concept in the food or agriculture sectors, there is a recent development in this area with novel aspects [92,93] Growing competitions and considerations for health concerns lead to increasing demand for sustainable and cost-effective production of food [94], and the development of more novel approaches for food packaging, coating, and processing to meet consumer’s demands [93,95]. In case of the emergence of nanotechnology in food packaging, as mentioned earlier, it should not be underestimated that targeted delivery of bioactive compound with reduced side-effects, stability of delivery systems [96,97], easiness in fabrication, regulation of food processed with nanomaterials, and public acceptance with affordable prices are highly encouraged to meet consumer’s demands, as well [98]. 

Manufactured nanomaterials such as thin films, nanotubes, and nanoparticles (made in one, two, and three-dimensional structures, respectively), have been extensively used in food sectors [97]. Usage of those aforementioned structures in food industries are categorized into food supplementations, food ingredients, food additives, food nutrients, and delivery systems (in the forms of nanoemulsions, nanoparticles, surfactant micelles, emulsion bilayers, and reverse micelles aimed at improvement in texture, color, and flavor). These structures are aimed at improving the taste or color of the food, and masking unpleasant tastes.

### 4.1. Antibacterial Activities

**Figure 2:**
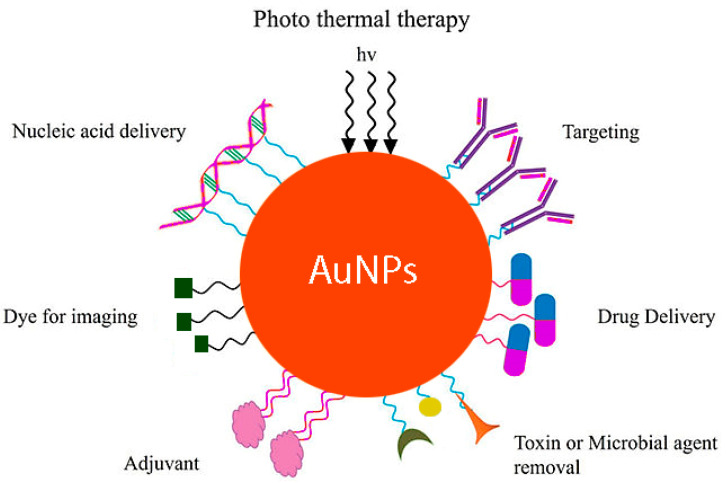
Functional properties of AuNPs.

In addition to the general properties of AuNPs [92,99], significant chemical properties of gold as a metal with the less corrosive one, and non-toxic effects [100,101], has attracted the focus of researches toward the usage of AuNPs in material based food industries (Figure 2). In a recent study, the function of 3-aminopropyltrimethoxysilane (APTMS) in the chitosan gold nanoparticles system was investigated. The addition of APTMS to chitosan resulted in a significant reduction in apparent viscosity, which can contribute to the achievement of higher miscibility in the structure. Additionally, the antimicrobial properties of a film composed of chitosan, APTMS, and AuNPs was evaluated against the Salmonella bacterium. The combined antibacterial activity of the three components was enhanced by their synergistic impact [102]. Anti-cancerous, anti-oxidant, and anti-microbial [103,104,105] properties of gold-based compounds, including anti-bacterial (especially against gram-negative bacteria when compared to Gram-positive bacteria [106]), anti-cancerous [41,107], anti-biofilm [108], anti-fungal (especially against *Aspergillus niger (A. niger)* and *Fusarium oxysporum*) [35,109,110,111], (majorly square-planar complexes of AuNPs [61]), anti-malaria, anti-amoebic, anti-leishmanial, and anti-trypanosomal features play an indispensable role in achieving success in food packaging, and prolongation of shelf life [16,103,110,112]. Mycelial extracts of the edible wild fungus *Cantharellus* sp. were used to manufacture AuNPs. On a variety of microorganisms, the antibacterial and antifungal capabilities of these NPs were evaluated. Significant antibacterial and antifungal properties were shown by the NPs [113]. Some studies tend to evaluate the effective ingredients responsible or in synergistic relations for induction of anti-bacterial effects in AuNPs against some bacterial strains such as: *E. coli*, *S. epidermidis*, and *Pseudomonas aeruginosa* [114]. Whereas, other studies recommend administration of other metals recruited as nanoparticles, including alloying colloidal silver-based nanoparticles with AuNPs. 

Results from one experimental study carried out by Yongwen Zhang et al. [115] aimed at the investigation of anti-bacterial effects of colloid AuNPs, indicated promising findings. Successful preparation of AuNPs, high dispersion stability, and excellent antimicrobial efficiency was carried out through an in situ reduction and stabilization of hyperbranched poly (amidoamine) with terminal dimethylamine groups (HPAMAM-N(CH3)2) in water, and an adjusted molar ratio of AuNPs (from 10 to 30) in feed [7,115]. HPAMAM-N(CH3)2 was used as an effective reducing and stabilizing agent. Anti-microbial effects of several strains, including *E. coli* (ATCC 8739), *S. aureus* (ATCC 6538), *B. subtilis* (ATCC 21332), and *Klebsiella mobilis* (K. *mobilis*) (ATCC 13048), *A. niger* (ATCC 16404), and *Penicillium citrinum* (*P. citrinum*) (ATCC 10499), were assessed by administration of 10^8^ CFU/mL from each bacterial cell suspension. Smaller particle size and narrower distribution were due to increased molar ratio and exacerbated anti-microbial effects (increased inhibition ratio up to ca. 98% at the low content of 2.8 μg/mL for AuNPs [115,116]).

To be more precise, AuNPs show less cytotoxic effects controversially compared with other metal-based nanoparticles such as silver [100,117]. They, as soluble complexes in organic solvents, have a strong binding affinity to other biological agents, which makes them more potentiated in releasing ions, and anti-microbial activities, as well [47,118].

Results from a multidisciplinary study conducted by Enrique Lima et al. [119] aimed at investigating the anti-microbial properties of AuNPs as biocide material against *E. coli* and *Salmonella typhi* after assessment of size, dispersion, and roughness of AuNPs. Those properties were supported by zeolite. Interestingly, they reported that there were anti-bacterial features by elimination of the aforementioned bacteria by Au-faujasite dispersed nanoparticles (particles sized 5 nm at the surface) [119].

An experimental study conducted by Saima Hameed et al. [120] was aimed at investigating the shape and concentration-dependent anti-microbial properties in AuNPs (with the size varying from 300 to 800 nm) against *E. coli*, *P. aeruginosa*, and *S. aureus*. Considered shapes for AuNPs were as following: nanospheres, nanostars, and nanocubes [120]. UV-vis spectroscopy, XRD, and TEM were used for the characterization and confirmation of AuNPs. AuNPs in the form of nanocubes depicted full ability in the inactivation of foodborne bacteria (zero survival percentage rate against all bacterial strains). Several cellular procedures, including cell loss, loosening of the cell wall, loss of flagella, releasing nucleic acid (or nucleic acid leakage), and loss of cellular matrix were reported after usage of those AuNPs through visual analysis (at lower concentrations). In this study, they concluded that despite similar surface areas in those AuNPs, anti-microbial interactions of gold nanoparticles with the Gram-negative or Gram-positive bacteria at lower concentrations will be shape-dependent [120,121].

Results from a preliminary interdisciplinary study carried out by Thirumurugan, A. et al. [122] demonstrated constructive effects of AuNPs exacerbated by incorporated food bio-preservatives (such as peptide of bacteriocin produced by *Lactobacillus plantarum* strain ATM11, and nisin) against reduction of microbial spoilage (*Micrococcus luteus*, *B. cereus*, *E. coli*, and *S. aureus*), that makes them proper to be used aimed at the prolongation of shelf life [122]. The most significant anti-bacterial activities were observed in the combinative usage of AuNPs/nisin, and AuNPs/bacteriocin against *E. coli*. They concluded that peptide-producing organisms presented more anti-microbial abilities against mentioned food-borne pathogens. Additionally, the combination of bacteriocins with AuNPs showed more anti-bacterial properties when compared to AuNPs/nisin [122].

Carlos H. Pagno and his colleagues [123], in their study, aimed at investigating anti-microbial properties of active biofilms of *quinoa* (*Chenopodium quinoa W.*) starch-containing AuNPs against *E. coli* (ATCC 25972), and *S. aureus* (ATCC 1901) [123]. Anionic silsesquioxane containing the 1,4-diazoniabicyclo, octane chloride group (a silica-based hybrid polymer) was used for stabilizing AuNPs [123]. The final product was in the form of AuNPs dispersed to biofilms of quinoa starch with enhanced tensile strength. The significant anti-microbial activity was reported as inhibition percentages of 99% against *E. coli*, and 98% against *S. aureus*. In addition to the significant anti-bacterial properties, and increased UV radiation absorption and a decreased solubility make them more proper for food packaging applications [123].

Sujan Chowdhury et al. [124] assessed the effects of poly(vinyl) alcohol cross-linked film containing AuNPs on prolongation in the shelf life of bananas. They compared acquired results to the incorporation of graphene oxide into poly(vinyl) alcohol cross-linked film. They reported that improved mechanical and physical properties (such as tensile strength, Young’s modulus value, water vapor transmission rate, and water solubility) are beholden to the dispersion of AuNPs and graphene oxide in the PVA cross-linked composites [124]. In case of advantages for anti-microbial efficacies, graphene oxide incorporated into poly(vinyl) alcohol showed less inhibition zone on disk diffusion agar tests against food-borne pathogens. They concluded that poly(vinyl) alcohol cross-linked film containing AuNPs was more efficient in increasing shelf life for bananas, and this efficacy in AuNPs was to a greater extent when glyoxal had been used as a cross-linker agent in poly(vinyl) alcohol cross-linked composite films. Mechanism of action for AuNPs against *E. coli* was cell lysis, which is accelerated by ethanol present in the poly(vinyl) alcohol film, electronic effects due to the changes in local electronic structures of the surfaces and their smaller sizes (less aggregated form and larger exposed expose a larger available surface area), as well [124].

Siying Tang et al. [125] designated their comparative study aimed at investigation of photocatalytic, and anti-microbial properties of gold-TiO_2_/Sodium alginate nanocomposite films (as a degradable film and adsorber for visible light), which were produced by hydrothermal and casting method. Mechanical properties of produced film containing AuNPs, conferred them good shape stability. In the case of assessed anti-bacterial properties, there was an improved antibacterial activity against *S. aureus* and *E. coli*. This activity is accelerated by synergistic effects SPR of AuNPs, and increased production of reactive oxygen species [125,126].

**Figure 3:**
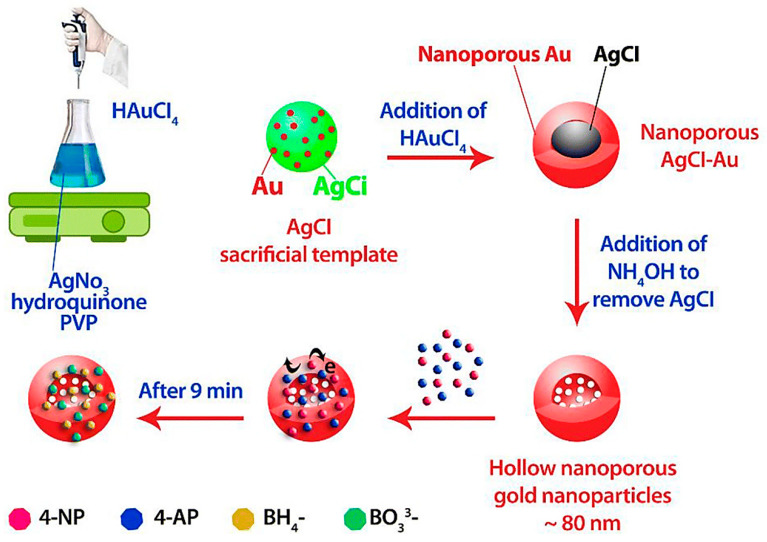
Chemical synthesis of AuNPs loading with different agents.

Biologically, those useful properties are highly dependent on ligands. Furthermore, migration of AuNPs due to high surface to volume ratio of AuNPs [51], production of radical oxygen (scavenging free radical species via catalytic reaction), hydrophobicity [52], ability to interact with both gram-negative and positive bacteria with adjusted size and morphology (even optimized concentration) [127,128,129] have been considered as a merit for their reaction with food-borne pathogens, making an impressive decrease in the incidence of bacterial pathogenesis in food, and subsequently in consumers. Figure 3, indicates that how the chemical synthesis of AuNPs affects their potential applications.

To be more specific, there are several strategies for enhancing the antimicrobial characteristics of AuNPs. These methods may be roughly classified into three groups, including chemical reduction, green synthesizing [11], and incorporation of AuNPs with other nanoparticles. Multiple research suggest that these properties are dependent on chemical agents bound to the AuNPs surface. Therefore, maximizing the antimicrobial activity of synthesized AuNPs is a controversial subject.

### 4.2. Barrier Properties

Permeability of water vapor, gases (e.g., CO_2_, O_2_, and N_2_), aroma chemicals, and light are barrier properties. The presence of moisture in food promotes microbial development and rapid food deterioration. Therefore, packaged food goods may have a much longer shelf life if they are wrapped in a film with enhanced barrier qualities (WVTR) [130]. Not only anti-microbial properties of AuNPs are of food packaging systems, but gas barrier properties also make them a proper choice. In general, there are not enough studies discussing the effect of AuNPs on barrier properties. However, similar to other metal nanoparticles, AuNPs depicted a notable improvement in barrier properties.

In a research by Pagno et al., it was reported that the presence of AuNPs does not negatively affect the water vapor permeability of quinoa starch film (*p* > 0.05). They also reported the improvement of permeability of oxygen and carbon dioxide after AuNPs were embedded within the biofilm (*p* < 0.01). The presence of AuNPs in the biofilms can significantly reduce the permeability of gases (*p* < 0.01), so that AuNPs, like other metal nanoparticles, are considered an additional barrier to the penetration of gases and consequently, due to the elongation and complication of the path within the film matrix, the penetration of gases leads to a delay in oxygen transfer. They also reported that increasing the AuNPs concentration from 2.5% to 5% did not change the permeability significantly (*p* > 0.05). In another study, AuNPs were embedded with a poly(vinyl) alcohol-based film [124]. It was reported that the addition of AuNPs enhanced the molecular structure tortuosity (*p* < 0.05), thus the water molecules are unable to pass through [45].

Barrier properties are reduced when a high concentration of AuNps are employed which the reason turns back to the agglomeration of AuNPs at higher concentration. It was shown that AuNPs can own distinct sizes (Table 2) and shapes which shows different behavior in agglomeration [131]. So, it is important to optimize the AuNPs concentration within the film matrix to reach the best barrier properties. Furthermore, the shape of AuNPs may affect the diffusion pathway which may result in better or worst barrier properties.

### 4.3. Antioxidant Properties

Metal and metal oxide NPs, carbon nanotubes (CNT), and different types of polymeric antioxidant NPs loaded with antioxidants demonstrated notable antioxidant properties. AuNPs as well as other common metal NPs own antioxidant potential. AuNPs are capable of interacting with both stable and unstable free radicals [132]. However, it was reported that AuNP’s potential as an antioxidant agent can be varied in a dose-dependent manner [133].

In a study, J. Beurton et al. [112], aimed to investigate the presser ing of controlled antioxidant properties (DPPH% and 2,2-azino-di(3-ethylbenzathiazolin-6-sulfinate) (ABTS•^+^) in the well-individualized I mobilized AuNPs on glass support (conducted through electrostatic interactions). As there was not any other peak except a localized SPR at 514 nm, no aggregation was reported. Promising results indicated a long-lasting antioxidant capacity characterized by a different kinetic (than colloidal AuNPs), and with the same efficiency. They designated the ability for prevention from the degradation of molecules (containing a thiol function) as a criterion for adaptable support of immobilized AuNPs. They represented that a ten-fold increase in the half-life of *N*-acetylcysteine, proved the efficacy of immobilized AuNPs for food packaging purposes with an adoptable covered surface, and the number of deposited AuNPs [112].

It has been reported that the high surface area to volume ratio of nanoparticles plays a vital role in their ability to scavenge free radicals [134]. Functionalized NPs attracted scientist’s attention in food packaging systems because of benefits such as unique and localization interactions of NPs with the bio components from the foods. In research carried out by Patra and his colleagues [64], AuNPs were synthesized by green synthesis using onion peel extract and named OP-AuNPs. Antioxidant activity was one of the main parameters under study. Based on the results, they reported that OP-AuNPs showed a notable DPPH scavenging capacity of 14.44%/100 µg/mL in comparison with 36.54%/100 µg/mL by butylated hydroxytoluene as a commercial antioxidant. OP-AuNPs also demonstrated higher radical scavenging compared to butylated hydroxytoluene. It was reported that the main reason turns back to the secondary metabolites, aromatic phenolic, and flavone groups in the onion peel extract and high surface area to volume ratio of nanoparticles [126,135].

### 4.4. Biosensing

One of the main concerns of customers is food quality and safety. Therefore, exploring fast, accurate, and efficient techniques to detect food quality is significantly important for human health. Biosensors depict a cutting-edge frontier in food packaging management [19], and nanomaterial such as AuNPs have been employed in the food contamination detection field to monitor, assess, and control food quality [136].

The intrinsic properties of AuNPs such as high stability, easy to control size and shape of the NPs, large surface-to-volume ratio, and excellent biocompatibility, offer themselves various benefits as sensors to make smart food packaging. For instance, a high surface/volume ratio provides high sensitivity and rapid responses. AuNPs also can be considered as multiple detection platforms [137]. Colorimetric sensors are one of the most common AuNPs-based sensors. AuNPs have shown their detecting potential in food contamination monitoring [137]. However, there are a few reports about employing AuNPs in food packaging systems.

M.R. Bindhu et al. [138] aimed to assess biosensory capabilities of gold and silver nanoparticles by a green approach (using *Solanum lycopersicum* extract). Carboxylic acid groups in the mentioned extract were recognized as reducing agents. UV-vis, FTIR, TEM, and energy dispersive spectroscopy (EDS) techniques were used for the characterization and confirmation of metal-based nanoparticles. Results of this study for monodispersed spherical gold, and silver nanoparticles showed significant SPR peaks at 546 nm and 445 nm, average size of 14 nm, and 12 nm, respectively. In addition to significant anti-microbial activity against *S. aureus* and *P. aeruginosa* existed in water samples, they were able to sense ions of heavy metals such as Fe^3+^ and Cu^4+^ in water by an SPR optical sensor, being efficient in water purification [131]. Results of this study were in agreement with other studies carried out by Govindhan Maduraiveeran et al. [132] indicating successes in the usage of AuNPs for sensitive detection of multi-target analytes with food safety and environmental monitoring purposes.

One innovative experimental study done by Cheuk-Fai Chow [139], was aimed at the investigation of correlations between the sensitivity of a colorimetric probe based on AuNPs (as a nano detector) to detection of microbial spoilage in meat products (stored at 4 °C), and volatile biogenic markers (such as dimethyl sulfide and histamine,). The ability of detection in the colorimetric probe is beholden to the ability in the transformation of the non-aggregated form of AuNPs to its aggregated form, after binding to aforesaid biomarkers. Of note, 0.5 and 0.035 μg/mL of AuNPs could detect dimethyl sulfide, and histamine biomarkers, respectively [139]. Additionally, they demonstrated that the constitution of AuNPs in the 2:1 H_2_O/DMSO mixture confers them to high stability to show the selective colorimetric response for mentioned biomarkers. In the case of microbial assessments, sensing signals of AuNPs for detection of mentioned volatile biogenic markers on 12 different raw meat samples were estimated. CFUs of 10^7^ were recognized as an alarm for the level of spoilage. For raw meat, preserved raw meat, raw fish, and raw crustaceans, UV-vis signals (at 520 nm) of 0.15, 0.1, 0.45, and 0.35 were reported alarm values, indicating harmful levels of food spoilage (CFUs of 10^7^).

The development of nanotechnology offers several industries, particularly the food packaging industry, significant prospects. As functional additives, several nanoparticles (such as nanoclay and metallic nanoparticles) have recently been incorporated to food packaging. Their beneficial impacts on modern packaging materials have been widely reported. According to the findings, the introduction of nanoscale fillers into the polymer matrix will alleviate packaging material difficulties while also enhancing functional properties [140]. These polymers incorporating nanocomposites have enhanced barrier characteristics, thermal properties such as melting temperature and glass transition, and altered functions such as surface properties and hydrophobicity. Inorganic nanoparticles have the ability to inhibit bacterial development inside packaging. By adding nanoscale components into biopolymer-based packaging composites, packaging-related waste may be decreased.

The migration behavior of nanomaterials from packaging may vary from that of conventional materials, and migrating nanoparticles may be more reactive and have a distinct toxicological profile. Thus, certain nanomaterials are prohibited in the EU due to insufficient toxicity data [141].

Multiple factors, such as temperature, time, the concentration of nanoparticles in polymer matrix, polymer characteristics, placement of the nanoparticles in the packaging material, interaction between the nanoparticles and the materials, sample selection type of material, contact type of material, and the nature of the food/food simulants, have been shown to influence the migration of nanomaterials from food packaging substance into food or food simulants. In general, nanoparticles have the ability to migrate into food, particularly when exposed to more acidic chemicals [142].

## 5. Hazard

Allergies and the release of heavy metals as the migration phenomenon are the two main safety concerns of nanoparticles. AuNPs depict a good safety profile. Considering AuNPs, as well as other metal nanoparticles have the potential of toxicity. AuNPs are capable of migration from packaging to food matrix and finally, they will be released in the human body after food consumption which is a toxin for different cells and tissues [138].

Currently, AuNPs are employed in active and smart food packaging systems at a relatively faster rate without desired knowledge and regulations, which can affect health and the environment. There is a likelihood of interacting between AuNPs and food molecules which leads to unwanted and toxic products. This issue is also important in smart food packaging systems which may result in false-positive detection [143].

Nevertheless, experimental use of AuNPs presented possible medical hazards as the surface to volume ratio causes catalytic properties and can make particles very reactive. Furthermore, nanoparticles easily pass cell membranes and can interact with intracellular metabolism [139]. Environmentally, AuNPs can be released into the ecosystem along with the nanocomposite. However, they are benign to the ecosystem if no accumulation happens in the environment. For instance, there is a report considering the non-toxicity of AuNps to zebrafish As at nano-scale gold-particles may exhibit size-related properties that differ significantly from the known properties of non-nano-scaled gold-particles, one cannot predict reliably the nature of AuNPs and a biologic system and interactions between AuNPs and living cells. Moreover, AuNPs showed different shapes with different charge and surface-chemistry and each one may have distinct behavior [144].

## 6. Future Prospective

To improve nano-packaging systems via AuNPs, it is necessary to consider all aspects including hazards. AuNPs as well as other common NPs, can migrate from packaging into the food matrix. However, AuNPs due to having various shapes can be a good candidate to modify a film structure with desired features.

Surface modification of the inner surface of the polymeric films (the surface which touches the food) can decrease the NPs diffusion by limiting the micro-channels. Creating a bilayered film, in which the inner layer is responsible for controlling NPs migration, can be a good solution. By the way, it is dictated that increasing the layers may consume more materials which can be considered as a hazard to the environment, so modifying the thickness of the layers is recommended.

As another recommendation, NPs modification can be another recommendation to decrease the rate of migration. Chemically, connection of NPs to the inner structure of the films can lower the migration phenomenon. It is also recommended that this connection, based on the various factors, can be permanent or temporary. Considering the previous point, AuNPs are known as good carriers. When AuNPs are loaded with an antibacterial agent, it is predicted to improve both barrier properties and migration rate.

As the final recommendation, AuNPs and ionic gold can show different activities against a range of microorganisms. It can be recommended for future studies to evaluate the level of improvement by employing both types of gold.

## 7. Conclusions

Food packaging is one of the most important challenges which has a direct effect on human health. In this regard, it is mandatory to engineer a packaging system that not only can increase foods shelf life but also can provide online information about food quality. Nanotechnology plays a vital role in this area of science. Metal NPs showed their potential in improving food packaging systems.

AuNPs with their unique potential demonstrated that can be integrated with other technologies in food packaging to achieve an active and smart food packaging system with suitable antibacterial, antioxidant, and scavenging properties while providing appropriate information about food quality. AuNPs have the potential to migrate from the film or coating packaging to the food matrix. Thereby, the way of polymeric film manufacturing, the concentration of AuNPs, employed agents, used biomaterial, type of food, and storage condition can affect AuNPs activity as antibacterial/antioxidant/biosensor agents.

## Figures and Tables

**Table 1:** Several recent studies evaluating on the usage AuNPs from plant sources.

Plant Source	Reducing Agents	Biological Effects	Microorganisms	Wavelength (nm)	Shape	NP Size (nm)	Year (Ref)
prunus cerasifera pissardii nigra leaf	extract	Anti-microbial and anti-fungal properties	*E. coli*, *S. aureus*, *B. subtillis*, *P. aeruginosa*, and *C. albicans.*	535	Spherical (20 nm)	20	2021 [69]
licorice root	extract	Antimicrobial and anticancer	*Bacillus subtilis*, *Staphylococcus aureus*, *Escherichia coli*, *Pseudomonas aeruginosa*, and *Salmonella typhi*	517	Spherical	53.7	2021 [70]
*Turnera diffusa* Willd	oplopanone, γ-eudesmol, hydroquinone-β-d-glucoside (arbutin) and inositol	antimicrobial properties and immune response	*Vibrio parahaemolyticus* and *Aeromonas hydrophila* *Longfin yellowtail*	540	multiple shapes, mostly spherical	24	2021 [71]
banana pith extract	Alkaloids, Flavonoids	antibacterial activity and catalytic reduction	*Bacillus subtilis*, *E. coli*, *Pseudomonas aeruginosa*	530–560	spherical	470	2021 [72]
*Ricinus communis* L.	alkaloids, terpenoids, steroids	antibacterial activity	*Bacillus cereus*, *Klebsiella pneumonia*, *Pseudomonas aeruginosa*, *B. cereus*	550	spherical	100	2021 [73]
Mentha Longifolia leaf	-	anti-human breast carcinoma	*breast carcinoma (Hs 578Bst)*	512	spherical shape particles	36	2021 [74]
curcumin	Curcuma pseudomontana isolated curcumin	antimicrobial, antioxidant, and anti-inflammatory activities, antioxidant and radical scavenging activities.	*Pseudomonas aeruginosa*, *Staphylococcus aureus*, *Bacillus subtilis* and *Escherichia coli*	542	spherical shape particles	20	2021 [75]
sing Pimenta dioica Leaves	extract	Photocatalyst, antioxidant, and antibacterial	*S. aureus* and *E. coli*	517	spherical shape particles	11	2021 [76]
Garcinia kola Pulp	extract	Antibacterial activity	*Staphylococcus epidermidis*, *Bacillus subtilis*, *Staphylococcus aureus*, *Escherichia coli*	564	spherical shape particles	18–38	2021 [77]
Kaempferia parviflora rhizome	extract	antimicrobial, antioxidant, and catalytic degradation agent	*Escherichia coli*, *Staphylococcus aureus*	540	spherical structure with high crystal in nature	20–60	2021 [78]
Clerodendrum inerme	extract	Antimicrobial, and antioxidant activities	*B. subtilis*, *S. aureus*, *Klebsiella*, and *E. coli*, *A. niger*, *T. harzianum*, and *A. flavus*	520	spherical	5	2020 [79]
Nigella arvensis leaf extract	flavonoids, alkaloids, and proteins	antibacterial, antioxidant, cytotoxicity against H1299 and MCF-7 cancer cell lines, and catalytic activities	*S. epidermidis*, *B. subtilis*, *S. aureus*, *E. coli*, *Serratia marcescens*, and *P. aeruginosa*	546	spherical shape mostly spherical in shape and less triangle, pentagon and hexagon shapes	3–37	2017 [80]
Aqueous Extract of Garcinia mangostana Fruit Peels	phenols, flavonoids, benzophenones, and anthocyanins	-	-	540–550 (UV-vis)	spherical shape particles	32	2016 [81]
Mimosa tenuiflora Bark Extract	deprotonation of hydroxyl groups present in polyphenolic molecules of extract	a moderate cytotoxic effect at 24 and 48 h was found Cytotoxicity on HUVEC cells using MTT, Cellular uptake, and catalysis 12.5 mg/L of Mt extract, we obtained a 50% inhibition (L50),		maximum in 280 nm and broad of 50 nm	multiple shapes	20–200	2019 [82]
Chenopodium formosanum shell extract	phenolic groups	antibacterial properties	*E. coli*, and *S. aureus.*	533	Most of the resultant Au NPs were spherical	8	2018 [83]
Leaf Extract of Ziziphus zizyphus	antioxidants, enzymes, and phenolic moieties	Antimicrobial and antifungal activity	*E. coli*, *S. marcescens* or *C. albicans*	peak in the range of 525–540 nm with a peak maximum in the range of approximately 527–535 nm	Majorly spherical and monodisperse	30–50	2018 [84]
Brazilian red propolis	hexane, dichloromethane and ethyl acetate, prenylated benzophenones	Antimicrobial, antifungal, and anticancer activities (dose-dependent cytotoxicity activities in bladder and prostate cancer cells)	*S. aureus*, *E. coli*, *S. mutans*, *C. albicans*	prominent peak at a range of 523–541 nm	mostly spherical shapes	8–15	2021 [85]
fresh peel (aqueous) extracts of Benincasa hispida	reducing enzymes as well as capping agents such as secondary metabolites	In vitro toxicity (antibacterial and anticancer)	Different Gram-positive and Gram-negative bacteria. Furthermore, the biosynthesized GNPs exerted remarkable in vitro cytotoxicity against human cervical cancer cell line, while sparing normal human primary osteoblast cells	a sharp absorption peak at 520 nm,	spherical in shape	22	2021 [86]
Platycodon grandiflorum leaf extract (Balloon flower plant)	flavonoids, saponins, alkaloids, amino acids, proteins, and carbohydrates	antipathogenic activity under optimal conditions	*E. coli* and *B. subtilis*	absorption at 545 nm	spherical in shape	15	2020 [87]
Hibiscus rosa-sinensis extract	alkaloids and flavonoids	-	-	520	spherical sized nanoparticles	16–30	2014 [88]
Galaxaura elongata (powder or free ethanolic) based extract		Anti-microbial properties	*E. coli*, *K. pneumoniae*, *S. aureus*, and *Methicillin-Resistant S. aureus*	520	spherical with a few rods, triangular, truncated triangular, and hexagonal AuNPs	3–77	2017 [89]
of Memecylon umbellatum leaf extract	Saponins, alkaloids, phytosterols, phlobatannins, phenolic compounds, phytosterols, and quinones	biocompatibility and anti-microbial activities	*B. subtilis*, *S. pneumoniae*, *S. aureus*, *S. typhimurium*, *Klebsiella aerogenes*, and *E. coli*	540	spherical-shaped nanoparticles	15–20	2018 [90]
Solanum nigrum leaf extract	phenolic compounds	strong DPPH radical and hydroxyl radical scavengers and antibacterial activity	*S. saprophyticus*, *B. subtilis*, *E. coli*, and *P. aeruginosa*	537	spherical-shaped nanoparticles and crystaline	50	2014 [91]

**Table 2:** AuNP dimension, weights, and volume.

AuNPs	Average Size (nm) (Diameter)	m_AuNP_ (10^−18^ g)	Volume (10^−25^ m^3^)
Citrate AuNS	12.5	19.7	10.2
Tryptophan AuNS	8.4	5.9	3.1
Tyrosine AuNS	9.9	9.8	5.1
CTAB/citrate AuNS	8.9	7.1	3.7
AuNS (CTAB)	77.9	4800	2475.2
AuNR (CTAB)	58.8 (length) 15.3 width	270	137.6
AuNPr (CTAB)	94.7 (side length)	1900	970.8
AuNC (CTAB)	47.5 (side length)	2100	1071.7

Cetyltrimethylammonium bromide (CTAB), spherical AuNP (AuNS), cubic AuNPs (AuNC), rod-shaped (AuNR), prismatic AuNPs (AuNPr).

## Data Availability

Not applicable.
